# Measurement of Gradient Strain Fields with Fiber-Optic Sensors

**DOI:** 10.3390/s23010410

**Published:** 2022-12-30

**Authors:** Valerii Matveenko, Natalia Kosheleva, Grigorii Serovaev, Andrey Fedorov

**Affiliations:** Institute of Continuous Media Mechanics, Ural Branch, Russian Academy of Sciences, 614018 Perm, Russia

**Keywords:** point fiber-optic sensors, fiber Bragg grating, distributed fiber-optic sensors, strain registration, strain gradient, Rayleigh scattering

## Abstract

The results of measuring gradient strain fields by embedded or mounted point fiber-optic sensors based on Bragg gratings and distributed fiber-optic sensors based on Rayleigh scattering are discussed. Along with the experiment, the results of numerical modeling of strain measurement errors associated with the assumption of uniaxial stress state in the area of the embedded Bragg grating and measurement errors by distributed fiber-optic sensors associated with gage length are presented. Experimental results are presented for 3D printed samples and samples made of polymer composite material. The geometry of the samples was chosen based on the results of numerical simulations, and provides different variants of non-uniform strain distribution under uniaxial tension, including the variant in which the derivative of the strain distribution function changes its sign. A good agreement of numerical results and experimental data obtained by distributed and point fiber-optic sensors in areas where the derivative of the strain distribution function keeps a sign and an increase in the error of strain measurement results by distributed fiber-optic sensors in areas where this derivative changes sign are demonstrated.

## 1. Introduction

Recent growth in the number of publications and applications related to new measurement technologies based on fiber-optic sensors (FOSs) is explained by a number of their advantages: low weight, small size, reliability, stability, resistance to external electromagnetic interference, high sensitivity, low power consumption, remote control and possibility to obtain real-time data [1]. FOSs provide the ability to measure various physical quantities: strain, acceleration, pressure, temperature and humidity [2]. The most widespread are point FOSs based on fiber Bragg gratings (FBGs), distributed fiber-optic sensors (DFOSs) based on Rayleigh, Brillouin and Raman scattering, and interferometric FOSs [3,4]. The FOSs, being small in size, can be embedded into material structure or fixed on its surface. These sensors find various applications, such as in civil structures [5,6], in aircraft structures [7], medical applications [8]. The development of fiber-optic sensors is associated with the use of various materials for the manufacture of optical fibers and various sensor manufacturing techniques, in particular the manufacture of Bragg gratings [9,10].

The simultaneous utilization of point FOSs and DFOSs in order to take the advantages of each of the sensor type is of significant interest. This allows to obtain a more complete picture of the mechanical state of the monitored object, based on FOSs measurement data. For example, point FOSs provide great capability for measuring dynamic strain, while DFOSs allow to measure static strain along the entire length of a fiber-optic cable. At the same time, there is a problem associated with differences in the readings of different types of sensors. Thus, for example, in [11] based on the results of measuring the strain and temperature in the reinforced concrete beams of a railway bridge by point FOS and DFOS for two and a half years, the possible reasons for the significant differences in the readings of the two types of sensors are given. Such reasons include: error in temperature compensation for DFOS, associated with not taking into account the temperature coefficient change along the optical fiber length, lower accuracy level of DFOS compared to point FOS, differences in connection conditions and a number of other reasons. In [12] a comparison of three types of sensors for groundwater temperature control is given: sensors based on FBG and DFOS based on Rayleigh and Raman scattering. The accuracy of temperature measurements of these sensors were evaluated based on the readings of the PT100 reference sensors. As a result of studies, it was shown that the required accuracy and spatial resolution for Raman scattering-based sensors can be achieved if continuous calibration of the sensors is ensured, while for FBG-based and Rayleigh scattering-based sensors the influence of strains acting on the sensor must be eliminated. A review paper [13] gives the results of studies in measuring corrosion in reinforced concrete by different types of FOSs. One result is the conclusion that DFOSs are preferable to point FOSs when monitoring corrosion propagation.

It is worth noting that when analyzing the measurement errors for the FOSs, as well as for other types of sensors that have the measurement basis dimension of the sensor, it is necessary to study the measurement results in the presence of the gradient of the measured quantity. In [14] it is described that FBG-based sensors do not have the capability of averaging strains over the gage length of the sensor. In the presence of strain gradients as a result of structural heterogeneity, the three-dimensional variant of strain in the Bragg grating area, the reflected spectrum does not always give distinct peaks and the reflection spectrum widens, which leads to errors in calculating strains based on the recorded physical quantities [15]. In [16], the characteristics of FBG-based sensors at different strain gradients and Bragg grating lengths were investigated in bending tests of an acrylic cantilever beam. As a result, the Bragg grating lengths were found for the corresponding strain gradients at which the reflected spectra have no split peaks. It should be noted that the results were obtained for isotropic materials and with a linear strain gradient.

In [17], a method of determining the distribution of strains, based on the results of measurements of the reflected optical spectrum from the FBG, is described. The developed method allows to calculate the influence of the strain fields on the reflection spectra of Bragg gratings. The authors of [18] tested this method experimentally and improved it for applications with high strain gradients. In [19] the measurement error of a distributed type of FOS based on Brillouin scattering and optical reflectometry in the time domain is discussed. Such questions as: why the initial strain gradient increases the error of measurement of the subsequent reading of the strain increment and how to estimate the magnitude of such an error are considered. The relationship between the strain gradient and strain measurement error is shown to affect the accuracy, which in turn affects the repeatability of measurements. A sectional shift method to minimize the error is proposed. Results of laboratory tests and studies show that the method can reduce the error by about 50% when the strain gradient is large. The influence of the combination of adhesive and protective coating properties on the accuracy of measurement results when using DFOSs glued to the surface of the object under study is noted [20]. Similar studies [21] were performed when measuring strains by DFOSs with polyamide coating embedded in reinforced concrete beams and glued to the surface of the concrete. As a result of the research, it was proposed to attach sensors to reinforcing bars using cyanoacrylate glue.

This paper presents analyses of the results of strain measurements by point FOSs based on FBG and DFOSs based on Rayleigh scattering embedded into material or fixed on surface of polymer composite material samples and samples obtained by additive technology. One of the distinctive features of the work is the measurement of non-uniform strain fields in subdomains where the derivative of the strain distribution function changes sign.

## 2. Samples with Gradient Strain Fields

This section provides information about the samples on which the studies related to the measurement of gradient strain fields by FOSs embedded into material or fixed on its surface were performed. Two types of samples were under investigation made of polymer composites and obtained by 3D printing technology. The shapes of the samples selected for testing were chosen based on the results of strain distribution patterns obtained by numerical simulations by the finite element method in order to select options with gradient strain fields acceptable for experiments.

**Figure 1:**
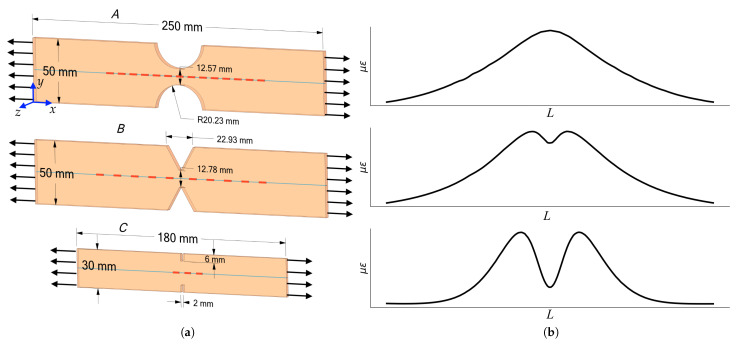
Specimen geometries (**a**) and its strain distribution along axial lines (**b**), respectively.

The geometries, samples sizes, and qualitative patterns of strain distribution along the axial lines of the samples under uniaxial tension are shown in Figure 1. Samples ***A*** and ***B*** consist of 20 layers of fiberglass prepreg and has 5 mm and 4.3 mm thickness, respectively. FOSs embedded in the material were placed along the axial line of the samples between the 10th and 11th layers. The assembly of the technological package for material pressing was carried out on a special tooling. The polymerization mode of the pressed technological package was carried out in a Langzauner press [22]. The sensors located on the material surface were glued with cyanoacrylate adhesive after the polymerization process was completed and the process package was removed. The characteristics of the obtained polymer composite material are determined by the following values: linear and shear moduli of elasticity $E_{xx}=26.5$ GPa, $E_{yy}=26.1$ GPa, $E_{zz}=6$ GPa, $G_{xy}=4$ GPa, $G_{xz}=3$ GPa, $G_{yz}=3$ GPa; Poisson’s ratios: $\nu_{xy}=0.144$, $\nu_{xz}=0.138$, $\nu_{yz}=0.18$. Embedded silica glass optical fibers have the following mechanical characteristics $E_{f}=71.4$ GPa; $\nu_{f}=0.17$, diameter 0.124 mm and a 0.012 mm thick protective coating, made of polyimide with mechanical characteristics $E_{c}=2.7$ GPa; $\nu_{c}=0.31$.

Sample ***C*** with a thickness of 3 mm was 3D printed with fused deposition modeling method from polylactide. The sample was made with the following technological parameters: nozzle diameter 0.4 mm, thickness of one layer 0.3 mm, temperature of the table and nozzle of the 3D printer 60 °C and 210 °C, respectively. The resulting 3D-printed material has a modulus of elasticity E=3.35 GPa and Poisson’s ratios ν=0.32. The process of printing samples with optical fiber embedded into material includes three steps. The first step is printing to the plane of the optical fiber location. In the second step, printing is paused and the optical fiber is laid and fixed. In the last step the printing is resumed until the sample is finished. Different fiber layouts are possible. One option is to keep the plane on which the fiber is laid flat. In this case the fixation of the fiber is carried out by the adhesive bonding. In the second option, when designing a model for printing, a cavity comparable to the diameter of the optical fiber is provided, in which the optical fiber is placed.

## 3. Calculation of Strains Based on Physical Quantities Recorded by Sensors

Two types of FOSs were used to measure strains: point FOSs based on the FBG and DFOSs based on Rayleigh scattering. The operation principle of point FOSs based on the FBG is relied on registration of the reflected part of the broadband optical spectrum launched into the optical fiber. The resonant wavelength of the reflected spectrum shifts when the Bragg grating is deformed. The relations between the physical quantities registered by the sensors (resonant wavelength of reflected spectra) and the strains in the Bragg grating area can be expressed [23].

(1) $$\begin{array}{c} \frac{\Delta \lambda_{1}}{\lambda^{*}}=\varepsilon_{3}-\frac{1}{2}n_{}^{2}\left(p_{11}\varepsilon_{1}+p_{12}\left(\varepsilon_{2}+\varepsilon_{3}\right)\right),\\ \frac{\Delta \lambda_{2}}{\lambda^{*}}=\varepsilon_{3}-\frac{1}{2}n_{}^{2}\left(p_{11}\varepsilon_{2}+p_{12}\left(\varepsilon_{1}+\varepsilon_{3}\right)\right), \end{array}$$

here $\varepsilon_{3}$ is the strain along the optical fiber, which is the result of the measurement by the sensor on the Bragg grating; $\varepsilon_{1},\varepsilon_{2}$ are principal strains in the plane perpendicular to the optical fiber; $\Delta \lambda_{1}=\lambda_{1}-\lambda^{*}$, $\Delta \lambda_{2}=\lambda_{2}-\lambda^{*}$ are the difference in the resonant wavelengths of the reflected spectrum in the deformed $\lambda_{1},\lambda_{2}$ and the non deformed $\lambda^{*}$ states; $p_{11}$, $p_{12}$ are strain-optic coefficients; *n* is the effective refractive index of the optical fiber in the zone of the fiber Bragg grating.

In the general case these relations do not have an unambiguous solution. For practical applications, the uniaxial stress state variant in the Bragg grating area is important. In this case, the relations between the axial components of the strain tensor are valid $\varepsilon_{1}=\varepsilon_{2}=-\nu \varepsilon_{3}$, where ν is the Poisson’s ratio of the optical fiber. Then relations (1) are reduced to one equation with one unknown:

(2) $$\frac{\Delta \lambda }{\lambda^{*}}=\left(1-\frac{n^{2}}{2}\left(p_{12}-\nu \left(p_{11}+p_{12}\right)\right)\right)\varepsilon_{3}\;$$

or

(3) $$\varepsilon_{3}=\frac{1}{k}\cdot \frac{\Delta \lambda }{\lambda^{*}}\;.$$

The sensors used in this work are based on silica glass optical fiber, which has $p_{11}=0.113$, $p_{12}=0.252$, n=1.458, ν=0.17 [24]. Thus, in the uniaxial stress state of the Bragg grating, the coefficient k∼0.78.

In general in the FOS, embedded into material or fixed on its surface using adhesive bonding, a complex-stress state will be realized. In this case, the assumption of uniaxial stress state in the Bragg grating area can be adopted to calculate strains based on relations (3). This assumption leads to inaccuracies in the calculation of strains based on the recorded physical quantities.

In this paper, the estimate of this error is based on the results of numerical simulations, which include the following steps [25]. For the sample model with embedded optical fiber under the considered loading case, the calculation of the stress–strain state is carried out by the finite element method based on the ANSYS software. The results of these calculations are strains $\varepsilon_{1}^{s},\;\varepsilon_{2}^{s},\;\varepsilon_{3}^{s}$ in the Bragg grating area and strains $\varepsilon_{x}^{m}$ in the material along the fiber, which must be measured in an experiment. The obtained values of strains in the Bragg grating area allow to obtain the values $\Delta \lambda_{1}/\lambda^{*}$, $\Delta \lambda_{2}/\lambda^{*}$ of the numerical model experiment on the basis of relations (1). Then, using the assumption of uniaxial stress state in the Bragg grating area and using $\Delta \lambda_{1}/\lambda^{*}$ and $\Delta \lambda_{2}/\lambda^{*}$ based on relations (3), two values of strain obtained from the sensor readings in the model experiment can be received:

(4) $$\varepsilon_{3}^{1}=\frac{1}{k}\cdot \frac{\Delta \lambda_{1}}{\lambda^{*}},\;\varepsilon_{3}^{2}=\frac{1}{k}\cdot \frac{\Delta \lambda_{2}}{\lambda^{*}}.$$

The difference between these values and the measured strain $\varepsilon_{3}^{m}$ gives an indication of the possible value of the error when using the assumption of uniaxial stress state in the Bragg grating.

In a real physical experiment, the reflected spectrum may be distorted and may not contain distinct peaks that allow the resonant wavelengths of the reflected spectra to be clearly recorded. In the implemented experiments, eight-channel HYPERION si255 interrogator by Micron Optics was used with an algorithm for selecting the resonant wavelengths by the center of gravity of the area under the main part of the reflected spectrum.

In addition to point FOSs, DFOSs based on Rayleigh backscattering have been used to measure strain. In contrast to FBG-based sensors, where only a small region of the optical fiber, where the grating is recorded, is sensitive to changes in strain and temperature, DFOSs use the entire length of the optical fiber as the sensing element. For distributed measurements Luna Innovations’ OBR 4600 high resolution optical backscatter reflectometer with distributed strain and temperature measurement is used. For measurements with a backscatter reflectometer, a section of the fiber to be analyzed is selected from a scattering trace of the optical fiber using specialized software. This section is divided into sub-areas of a given length (gage length) with a certain step (distance between sensors), which can be treated as individual sensors. The sub-areas may overlap and the center of each sub-area is one of the points on a diagram of distributed strain or temperature measurements. External influences on the optical fiber (strain, temperature) cause a shift of the signal in the frequency domain of the analyzed fragment relative to the reference one. To obtain strain or temperature, the spectral shift is calculated between loaded and reference states and multiplied by the strain or temperature sensitivity coefficient of the optical fiber.

The spectral shift (Δν) of the measured region of the optical fiber is analogous to the shift in spectrum or resonant wavelength shift (Δλ) of a Bragg grating and is related to changes in strain and temperature as follows:

(5) ΔλΔλλλ=−ΔνΔνν=ν=Kε·ε+KT·ΔT,

where $K_{T}$ and $K_{\varepsilon }$ temperature and strain coefficients. For most optical fibers with a germanosilicate core $K_{T}=6.45\cdot 10^{-6}$ C^−1^, $K_{\varepsilon }=0.78$.

With constant strain of the optical fiber section to be measured, the temperature change can be expressed as:

(6) $$\Delta T=-\frac{\bar{\lambda }}{cK_{T}}\Delta \nu ,$$

where $\bar{\lambda }$ is central scanning wavelength, *c* is speed of light.

Similarly, the temperature-compensated strain variation can be expressed in terms of:

(7) $$\varepsilon =\frac{\bar{\lambda }}{cK_{T}}\left(\Delta \nu^{T}-\Delta \nu \right),$$

where $\Delta \nu^{T}$ is spectral shift measured at a specific section of the optical fiber which does not experience strain variation.

## 4. Results of Strain Measurement in Samples with Gradient Strain Fields

In the tensile tests, strain was measured in samples ***A*** and ***B*** using FBG-based sensors and DFOS based on Rayleigh scattering embedded into material or fixed on its surface. Figure 1 shows the FBG sensor locations, which were 5 mm in length. Strain measurements by DFOSs were carried out with a spatial resolution of 2 mm, where each point was calculated on a 10 mm base.

To estimate the error of using the assumption of uniaxial stress state in the Bragg grating area in calculating strains based on physical quantities recorded by sensors em-bedded into samples ***A*** and ***B***, calculations for the numerical experiment described above have been performed.

For sample ***A*** in the area of maximum strain in the material $\varepsilon_{x}^{m}/P=1.52\cdot 10^{-10}$ N^−1^, and the calculated physical quantities have the following values: $\Delta \lambda_{1}/\lambda^{*}=0.000117$, $\Delta \lambda_{2}/\lambda^{*}=0.000118$. At these values, the strain $\varepsilon_{3}^{1}$, $\varepsilon_{3}^{2}$ that differ from the measured strain $\varepsilon_{x}^{m}$ by no more than 1.3% are obtained using relations (4). Similar estimates were obtained for sample ***B***. These numerical results show a small error from the use of the assumption of uniaxial stress state in the Bragg grating area. It should be noted that this error is determined not only by the loading variant of the sample, but also by the ratio of the mechanical characteristics of the material and fiber.

**Figure 2:**
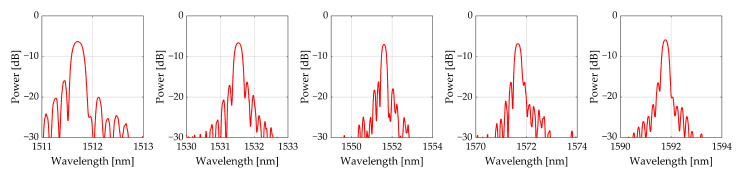
Reflected spectra of FBG for sample ***B***.

The numerical results showing the closeness of the values $\Delta \lambda_{1}/\lambda^{*}$ and $\Delta \lambda_{2}/\lambda^{*}$ are confirmed by the patterns of reflected optical spectra, which have quite pronounced single peaks. An example of these spectra for sample ***B*** is shown in Figure 2.

**Figure 3:**
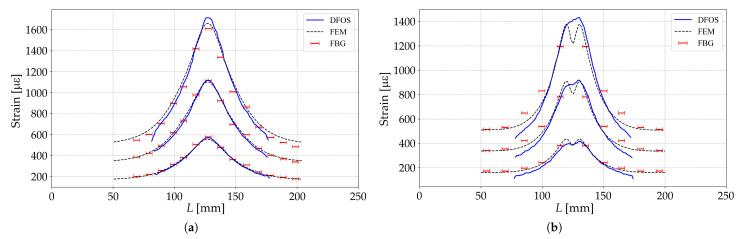
Strain values along the center line obtained by point FBG-based sensors, DFOSs measurements and FEM calculations for samples ***A*** (**a**) and ***B*** (**b**).

Figure 3 shows the strain distributions along the length of samples ***A*** and ***B*** at loads P=0.9, 1.8, 2.8 kN, when measured by FBG-based sensors (shown with red bars with length equal to the length of the sensor), DFOSs based on Rayleigh scattering (shown with blue line) and numerical results based on finite element method (FEM) (shown with black dashed line). In the results shown, the FBG-based sensors were embedded into material and DFOSs were glued to the surface with cyanoacrylate adhesive.

In general, the obtained results show a good agreement between the strain values obtained by the DFOSs and those obtained from the finite element calculations. The greatest divergence between the strain measurement results of the DFOSs and the numerical analysis results occurs at areas where the derivative of the strain distribution function changes sign.

The results obtained on the basis of FBG differ from the numerical results obtained by the finite element method by 12%. One of the reasons for these differences could be the non-uniform distribution of strain. For the FBG, along the length of which the strain distribution is non-uniform, the following relation is valid for the shift of the resonant wavelength of the reflected spectrum:

(8) $$\begin{array}{c} \frac{\Delta \lambda_{1}}{\lambda^{*}}=\frac{1}{L}\int_{z=0}^{z=L}\left\{\varepsilon_{3}-\frac{1}{2}n_{}^{2}\left(p_{11}\varepsilon_{1}+p_{12}\left(\varepsilon_{2}+\varepsilon_{3}\right)\right)\right\}dz,\\ \frac{\Delta \lambda_{2}}{\lambda^{*}}=\frac{1}{L}\int_{z=0}^{z=L}\left\{\varepsilon_{3}-\frac{1}{2}n_{}^{2}\left(p_{11}\varepsilon_{2}+p_{12}\left(\varepsilon_{1}+\varepsilon_{3}\right)\right)\right\}dz, \end{array}$$

where *L* is the length of the FBG, *z* is the coordinate along the length of the FBG. The structure of this ratio demonstrates the effect of the strain gradient on the magnitude of the resonant wavelength of the reflected spectrum.

**Figure 4:**
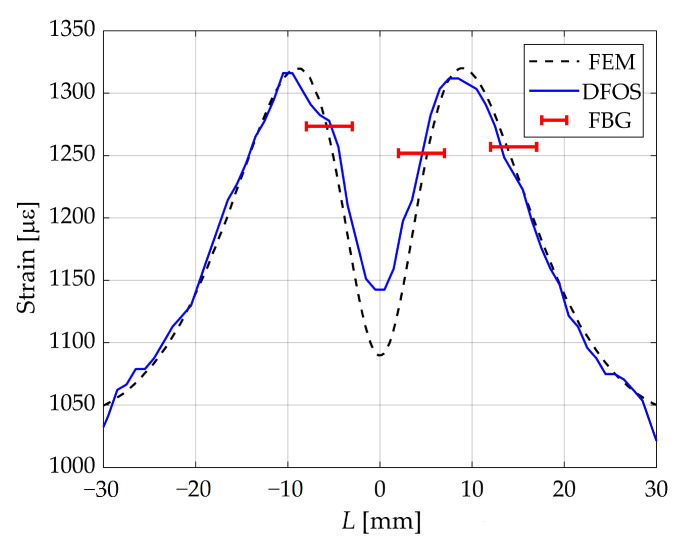
Strain values along the central axis line obtained by measuring with point sensors (FBG), DFOSs and FEM calculations.

One of the key factors determining the accuracy of measuring an non-uniform strain field is the value of the gage length. To study the influence of this factor, taking into account that the greatest discrepancies for samples ***A*** and ***B*** of the measurement results by DFOSs with the results of numerical modeling takes places in the zones of variation of the sign of the derivative of the strain distribution function, experiments were performed to measure strain by point and distributed FOSs embedded in sample ***C***. The geometry of this sample was chosen taking into account the study of the results of measuring strain in zones where the derivative of the strain distribution function changes sign. On the basis of numerical modeling by the finite element method, a variant in which the dependence of strain under tension of the sample has two pronounced peaks at a distance of 20 mm was considered. For this sample under uniaxial load of 300 N Figure 4 shows the results of measuring strain by sensors based on FBG having a length of 5 mm (shown with red bars with length equal to the length of the sensor), DFOSs based on Rayleigh scattering with a sensor base of 5 mm and a spatial step of 1 mm (blue line) and the results of numerical simulation by the finite element method (black dotted line). The results in Figure 4 are shown for FOSs embedded in the material. Comparison of the measurement results by DFOSs embedded in the material and fixed on the surface with cyanoacrylate glue demonstrate a difference in strains within 6.7%.

The distributed strain measurement is carried out by registering Rayleigh scattering profiles at the initial, unloaded moment of time and in the loaded state. Then the scattering profiles are divided into fragments of a given length along the measured section of the optical fiber, which are transferred using the Fourier transform to the frequency domain, where a cross-correlation procedure is performed for each section to determine the spectral shift [26].

The length of the fragments Δx (gage length) into which the scattering profile is divided affects the accuracy of measurements. As the gage length increases, the number of points on the scattering profile increases, which are used to transfer data from the time domain to the frequency domain, and, consequently, the spectral resolution increases. On the other hand, the presence of a significant strain gradient along the gage length makes it difficult to calculate the cross-correlation peak [27]. Thus, the most optimal gage length is limited on the one hand by the smallest number of points on the scattering profile that are necessary to obtain a spectrum in the frequency domain (*a*), and on the other hand, by the maximum length of the fragment (*b*), on which, in the presence of a strain gradient, a cross-correlation algorithm is possible:

(9) a≤Δx≤b.

**Figure 5:**
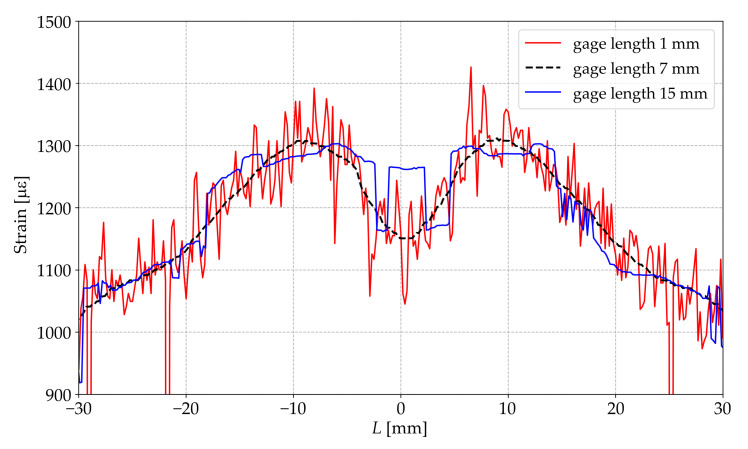
Strain distributions along the central axial line obtained by DFOS at different gage lengths.

Going beyond the designated limits leads to a high signal-to-noise ratio (SNR) and significant errors as shown in Figure 5, which presents the graphs of the strain distribution along the sample measured by DFOS with Δx=1mm<a, Δx=15mm>b and a<Δx=7mm<b.

**Figure 6:**
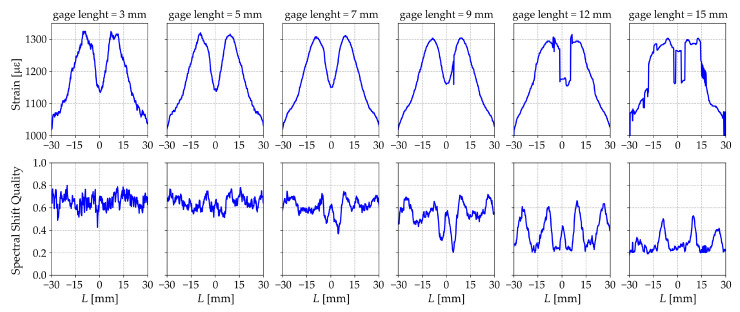
Strain distribution and Spectral Shift Quality for different gage lengths along the sample ***C***.

Based on the obtained experimental results, values of *a* in the range of 3÷5 mm can be recommended. To estimate the upper limit value *b* of the gage length, it is possible to use the Spectral Shift Quality parameter to assess the quality of the performed cross-correlation procedure.

(10) $$\mathrm{Spectral}\;\mathrm{Shift}\;\mathrm{Quality}=\frac{\max(U_{j}\left(\nu \right)★U_{j}\left(\nu -\Delta \nu_{j}\right))}{\sum U_{j}{\left(\nu \right)}^{2}},$$

where $U_{j}\left(\nu \right)$ is the spectrum of a given data fragment in the initial unloaded state; $U_{j}\left(\nu -\Delta \nu_{j}\right)$ is the spectrum for this fragment in the deformed state; and ★ is the cross-correlation operator. Spectral Shift Quality varies in the range [0, 1], where 1 is the ideal correlation [27]. Figure 6 shows that the Spectral Shift Quality parameter for a gage length bigger than 9 mm is low in the areas of the greatest strain gradient.

In addition to the distortion of the measurements associated with the features of the algorithm for calculating distributed strain, with an increase in the gage length, it becomes difficult to determine local areas of non-uniform strain distribution, due to averaging the readings. This is most pronounced in zones where the derivative of the strain distribution function changes sign.

The introduction of an error by averaging the measurements is demonstrated in a numerical example, where the distribution of strain along the sample obtained by the finite element method is taken as the initial true values (the initial function). The values of strain distributions with different gage lengths are obtained by applying a simple moving average function with a window length corresponding to the gage length

(11) $$SMA_{t}=\frac{1}{n}\sum_{i=0}^{n-1}p_{t-i}=\frac{p_{t}+p_{t-1}+\ldots +p_{t-i}+\ldots +p_{t-n+2}+p_{t-n+1}}{n},$$

where $SMA_{t}$ is the value of the simple moving average at point *t*; *n* is the number of values of the original function for calculating the moving average; $p_{t-i}$ is the value of the original function at point (t−i).

**Figure 7:**
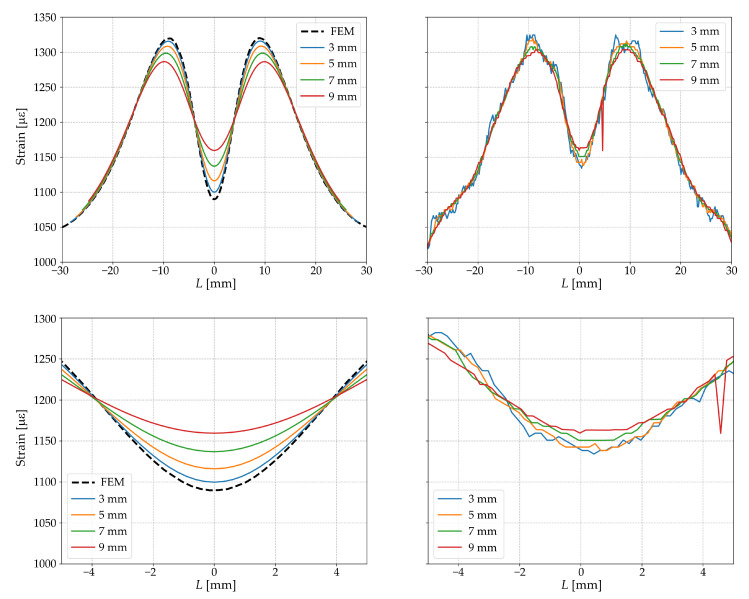
Strain distributions along the central axial line of sample ***C*** with different gage lengths obtained by FEM (left column) and DFOSs (right columns).

The obtained distributions with different gage lengths are shown in Figure 7. These results demonstrate that the error in measuring strain depends on the length of the sensor (gage length) and decreases asymptotically with decreasing gage length. This conclusion is most pronounced in the zones where the derivative of the strain distribution function changes its sign. The results of the numerical experiment qualitatively confirm the experimental data obtained for the value Δx.

## 5. Conclusions

The geometry of the samples is presented, which provides various variants of the gradient distribution of strain under uniaxial tension, including variants with a change in the sign of the derivative of the strain distribution function. To produce samples of the selected geometry two types of materials with embedded sensors were used: polymer composite materials obtained by direct pressing method and polymer materials obtained by 3D printing. A comparison of the results of measuring strains by point sensors based on Bragg gratings and DFOSs based on Rayleigh scattering embedded into material or fixed on its surface and the results of numerical simulation by the finite element method is presented. Simulation results related to the estimation of the error in calculating strains based on the results of physical quantities recorded by point and distributed FOSs are presented. A good agreement between the results of numerical modeling and the results in the areas where the derivative of the strain distribution function retains the sign and differences in the areas where this derivative changes the sign is demonstrated. The result of these comparisons is explained on the basis of numerical modeling. All data necessary to reproduce the above results are given in the paper.

## Data Availability

Not applicable.
